# Printing Defect Detection Based on Scale-Adaptive Template Matching and Image Alignment

**DOI:** 10.3390/s23094414

**Published:** 2023-04-30

**Authors:** Xinyu Liu, Yao Li, Yiyu Guo, Luoyu Zhou

**Affiliations:** 1 Electronics and Information School, Yangtze University, Jingzhou 434023, China; 2 Institute for Artificial Intelligence, Yangtze University, Jingzhou 434023, China

**Keywords:** printing defect detection, feature map cross-correlation (FMCC), image alignment, template matching

## Abstract

Printing defects are extremely common in the manufacturing industry. Although some studies have been conducted to detect printing defects, the stability and practicality of the printing defect detection has received relatively little attention. Currently, printing defect detection is susceptible to external environmental interference such as illuminance and noise, which leads to poor detection rates and poor practicality. This research develops a printing defect detection method based on scale-adaptive template matching and image alignment. Firstly, the research introduces a convolutional neural network (CNN) to adaptively extract deep feature vectors from templates and target images at a low-resolution version. Then, a feature map cross-correlation (FMCC) matching metric is proposed to measure the similarity of the feature map between the templates and target images, and the matching position is achieved by a proposed location refinement method. Finally, the matching image and the template are both sent to the image alignment module, so as to detect printing defects. The experimental results show that the accuracy of the proposed method reaches 93.62%, which can quickly and accurately find the location of the defect. Simultaneously, it is also proven that our method achieves state-of-the-art defect detection performance with strong real-time detection and anti-interference capabilities.

## 1. Introduction

Defect detection has always been the focus of research in computer vision [1,2]. Nowadays industrial printing has become a necessity that fills our daily lives in various forms, such as books, newspapers, advertisements, packaging boxes, and so on. At the same time, the rapid development of industrial printing has also brought a series of printing quality problems [3]. Common printing defects are mainly presented in two forms. The first form is color defects, including uneven printing ink, deviation of printing color, distortion of printing color, etc. The second one is shape defects, including incomplete printing, offset position of printing fonts, distortion of printing patterns, etc. Therefore, how to quickly and efficiently detect printing defects is the focus of the current manufacturing industry. The initial printing defect detection mainly relies on manual visual detection, which requires a lot of manpower and material resources. Moreover, there are uncontrollable shortcomings such as slow speed and low precision. To solve these problems, computer vision technology has been introduced for printing defect detection [4].

As far as we know, defect detection methods are one of the most popular research topics in computer vision [5,6,7]. Although some studies have been conducted to detect printing defects, the stability and practicality of printing defect detection has received relatively little attention. At present, printing defect detection is mainly implemented based on template matching [8]. Template matching refers to finding the regions similar to the given template in the target image. Template matching has always been the focus of research in image processing, which has been applied in many fields, such as object detection [9], object tracking [10], defect detection [11] and so on. Traditional template matching is proposed based on pixel-level methods [12]. These algorithms include sum of squared differences (SSD) [13], zero-mean normalized cross-correlation (ZNCC) [14], sum of absolute differences (SAD) [15], etc. They often cost a lot of time to implement matching and can not achieve real-time detection. In addition, they do not show strong robustness in complex scenes, which limits their application. In real scenarios, the target image will be inevitably contaminated by environmental interference, including illumination and noise. Therefore, traditional template matching cannot satisfy practical industrial requirements.

In order to solve these limitations, researchers have proposed some methods based on deep learning. With the rapid development of artificial intelligence, deep convolutional neural networks (CNNs) have become a dominant direction in various fields, including image inpainting [16], object tracking [17], image segmentation [18], and so on. In recent years, there is a two-stream structure based on CNN, called the Siamese network [19], which can also be viewed as a matching problem. Inspired by the Siamese network, a neural network was introduced to measure the similarity between the target image and the template, which showed better anti-interference ability under complex scenes. The common method was based on a parameter-free robust best buddies similarity (BBS) method [20], which combined with a neural network for nearest-neighbor (NN) matching by extracting location and shape information. Considering that the samples may be deformed, a deformable diversity similarity (DDIS) method [21] was introduced to measure similarity by finding the features of potential matching locations. In addition, in order to overcome the interference of the external environment, such as illumination and noise, etc., Fang et al. [22] proposed a smart reinforcement learning (RL) method. This method could learn to tune parameters automatically to enhance model performance, which could provide a broader perspective on overcoming the interference and reference for template matching.

Motivated by the successes of deep learning, this paper proposes a printing defect detection method which is composed of two modules, including a template matching module and an image alignment module. The main contributions of this paper are given as follows:

(1) A scale-adaptive deep convolutional feature extraction method is proposed for template matching. Moreover, the feature extraction is implemented on a low-resolution version of the template and the target image. Therefore, the method effectively decreases the matching time.
(2) A feature map cross-correlation (FMCC) matching metric is proposed to measure the similarity between the feature map of the template and the target image. The introduction of a matching metric can greatly improve the accuracy of the similarity measurement.
(3) Furthermore, an image alignment and difference detection module is introduced to adjust the defect position and greatly improve the effect of defect detection. Therefore, the proposed method can obtain state-of-the-art detection performance with strong real-time performance and anti-interference capabilities.

The rest of the paper is organized as follows. In Section 2, we present the related works on printing defect detection. Then, our proposed method is detailed in Section 3, while Section 4 presents the experimental results. Finally, the conclusion is given in Section 5.

## 2. Related Work

Over the past few years, the manual detection of printing defects can no longer meet strict artistic effects and quality pursuit. Computer vision methods have made some progress in defect detection. However, the current printing defect detection technology is subject to certain technical limitations, and there is still a lot of research space in terms of accuracy and speed.

In recent years, researchers have proposed some computer vision-based defect detection methods [23]. Golnabi et al. [24] proposed a printing defect detection method based on a three-dimensional spatial coordinate system, which used the pixel indexes in the three-dimensional coordinate system to achieve defect detection. The experimental results showed that the method can detect defects in complex printed matters, but its robustness was poor. For defect classification problems, Luo et al. [25] combined a BP neural network with an image histogram to achieve defect classification and detection. The system can extract the defects of small-format printed matters and accurately classify printed matter images, but it needs special hardware equipment and has poor applicability. Meanwhile, under the premise of ignoring unimportant pixels, Salahdine et al. [26] proposed a defect detection method based on a dynamic threshold to shorten the detection time. The simulation results showed that the algorithm was fast and could output multiple parameters. Considering that noise also affects the detection results, Tian et al. [27] proposed an adaptive filtering denoising algorithm, which could effectively avoid the influence of noise without damaging image information.

Template matching is the core technology of the defect detection method. It searches the best matching region in the target image according to the given template. In order to accurately achieve the matching region with the highest similarity of the template, it is usually necessary to define a similarity measurement metric to measure the similarity between the target image and the template. Some traditional template matching metrics are proposed based on the pixel level, and thus they cost much time to implement the matching. Moreover, their matching performance will become worse when the image is contaminated with external interference, such as illuminance and noise.

In recent years, several robust algorithms combined with neural networks have been proposed for template matching and defect detection. Based on the concept of bi-directional similarity (BDS), a parameter-free and robust method was proposed based on best buddies similarity (BBS) method [28]. It does not directly take the actual distance value but achieves the bidirectional matching region by counting the number of best buddies pairs (BBPs), which can improve robustness. Moreover, in order to improve computational efficiency and make better use of neural networks, a deformable diversity similarity (DDIS) matching method was proposed based on the nearest-neighbor (NN) method. This method not only considers complex deformations but also improves the accuracy of localization. However, the DDIS algorithm has difficulty dealing with the problem of scale change. To accommodate matching patterns in non-single scenarios, Cheng et al. [29] proposed a quality-aware template matching (QATM) method. This method can perform many matching modules, including one–one, one–many, and many–many. However, they perform poorly in complex industrial environments. In this paper, we propose an improved template matching algorithm which achieves better matching results compared to previous methods. Moreover, an image alignment module is introduced to adjust the defect position and achieve state-of-the-art detection performance.

## 3. Methods

**Figure 1:**
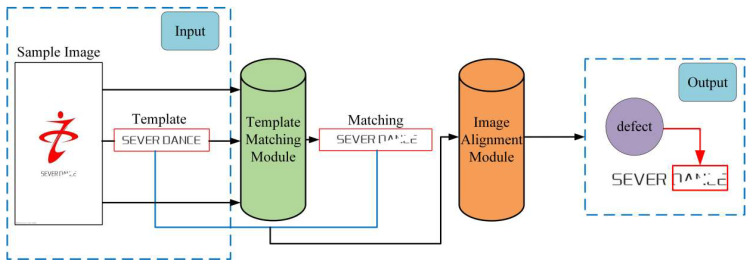
Schematic diagram of the printing defect detection based on scale-adaptive template matching and image alignment.

To achieve efficient and reliable printing defect detection, we propose a printing defect detection method based on scale-adaptive template matching and image alignment. As shown in Figure 1, the schematic diagram consists of two modules: a scale-adaptive template matching module, and an image alignment module. The details of the method will be presented in this section.

### 3.1. Template Matching Module

**Figure 2:**
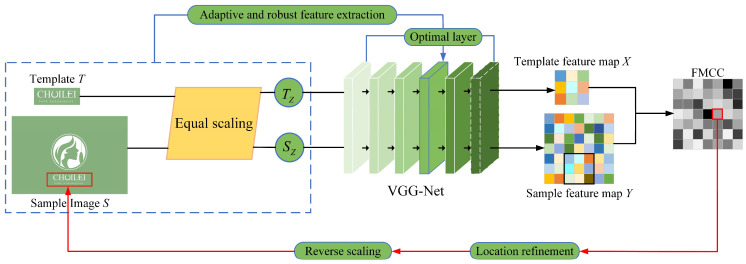
Schematic diagram of the improved template matching module, including a scale-adaptive deep convolutional feature extraction method and FMCC-based similarity measurement method.

Aiming at the shortcomings of the existing template matching algorithms, this paper proposes a template matching algorithm based on a scale-adaptive feature extraction method with low-resolution images. Moreover, a novel feature map cross-correlation (FMCC) matching metric is introduced to measure similarity in the matching processing. As shown in Figure 2, the proposed template matching module consists of two parts, including a scale-adaptive deep convolutional feature extraction method and an FMCC-based similarity measure method.

#### 3.1.1. Scale-Adaptive Deep Convolutional Feature Extraction Method

In order to better describe our proposed template matching module, the sample (target image) *S* and the template *T* are given explicit spatial domain parameters. We suppose the sample $S\in I^{a\times b\times 3}$, where *a* and *b* represent the width and height of the sample, respectively, and the template $T\in I^{w\times h\times 3}$, where *w* and *h* represent the width and height of the template, respectively. Our method does not directly use the traditional sliding window to search for the sample. We introduce CNN to extract feature vectors of different depths. Furthermore, our feature extraction method neither specifies the size of the input image nor specifies the layer of depth features. Instead, the proposed method can adaptively identify the optimal layer in the CNN, and thus has superior robustness.

The scale-adaptive feature extraction method proposed in this paper will be introduced in detail below. We use VGG-Net [30] as the feature extraction network and do not specify the size of the sample *S* and the template *T*. For different images, VGG-Net is introduced to adaptively identify the optimal layer and extract the depth feature vectors. For CNNs, the feature map output by each layer has its corresponding receptive field, and the receptive field of the *l* layer is defined as:

(1) $$rf_{l}=\{\begin{array}{cc} rf_{l-1}+(\left(f_{l}-1\right)\cdot \prod_{i=1}^{l-1}s_{i}) & l>1,\\ 3 & l=1, \end{array}$$

where *rf_l_* represents the receptive field of *l* layer, *rf_l_*_-1_ represents the receptive field of the previous layer, *f_l_* is a filter size of the *l* layer, and *s_i_* represents a stride. Here, 3 is the initial receptive field of the first convolution layer. If the receptive field of the template is smaller than the receptive field of the optimal layer, the layer will fill some meaningless areas with zeros. Therefore, we limit the optimal layer to have a receptive field which is smaller than or equal to the template. Here, the constraint of the receptive field is detailed as:

(2) $$l^{\infty }=\max(l-k,1)\;\;\;rf_{l}\leq \min(w,h)$$

where *k* is an integer greater than or equal to 0. Notice that we specify the amount of padding for the optimal layer receptive field that needs to be filled with zeros. For example, if a layer has a 5 × 5 receptive field and the stride of the filter is 2, the number of zeros to be filled in the optimal layer is 5 + 2*d*, where *d* is an integer greater than or equal to 0. Compared with the sliding window method, we only compute feature vectors once for each sample and template with CNN, which greatly decreases the number of parameters and shortens the operation time.

#### 3.1.2. FMCC-Based Similarity Measure Method

To effectively decrease the matching time, the feature extraction method is implemented on a low-resolution version of the template and the sample. Our method first sets the scale zooming factor *Z* = 2*^r^* (*r* = 0, 1, 2, ……) to scale down the template *T* and the sample *S* in equal proportions. Then, the scaled sample *S_z_* and the scaled template *T_z_* are both sent into feature extraction network. Finally, the optimal layer is adaptively obtained, and feature map *X* (the template) and feature map *Y* (the sample) are extracted from the optimal layer. In addition, we also keep the sample *S* and the template *T*, which can be mapped to high resolution images after matching and easily embedded into different scenarios. For the extracted feature map, we measure the similarity between *X* and *Y* by FMCC, and FMCC is detailed as:

(3) $$FMCC_{i,j}=\frac{<X,\;\bar{Y}>}{\left|X\left|\right|\bar{Y}\right|}$$

where < > denotes the inner product of *X* and *Y*. Inspired by traditional template matching algorithms, our proposed FMCC matching metric also adopts the sliding window method. However, our method is performed on the feature map, which can greatly reduce the computational cost of sliding the window directly on the image. The FMCC metric first extracts a feature patch $\bar{Y}$ from the sample feature map *Y*. The size of the feature patch $\bar{Y}$ is the same as the template feature map *X*. Then, a convolution method is used to calculate the location (*u*, *v*) which has the maximum FMCC. Finally, according to the location obtained from the feature map, it is mapped to the sample image through back-projecting, and the obtained regions are the result of template matching.

Moreover, we also use location refinement algorithms to improve matching accuracy. Using the FMCC as the weight, the maximum location obtained on the feature map is mapped back to the region of the sample image by a weighted sum. Firstly, we set the position of the initial box obtained on the sample image as $\left(\left(x_{1}^{*}{,\;y}_{1}^{*}\right),\left(x_{2}^{*}{,\;y}_{2}^{*}\right)\right),$ where $\left(x_{1}^{*}{,\;y}_{1}^{*}\right)$ is the upper-left location of the initial box, and $\left(x_{2}^{*}{,\;y}_{2}^{*}\right)$ is the bottom-right location of the initial box. Then, the position of the box after refinement is obtained as $\left(\left(x_{1}{,\;y}_{1}\right),\left(x_{2}{,\;y}_{2}\right)\right),$ which is transformed as follows:

(4) $$x_{1}=\frac{\sum_{m=-1}^{1}\sum_{n=-2}^{1}FMCC_{u+m,v+n}\cdot \left(x_{1}^{*}+n\cdot \prod_{i=1}^{l^{\infty }-1}s_{i}\right)}{\sum_{m=-1}^{1}\sum_{n=-2}^{1}FMCC_{u+m,v+n}}$$

(5) $$y_{1}=\frac{\sum_{m=-1}^{1}\sum_{n=-2}^{1}FMCC_{u+m,v+n}\cdot \left(y_{1}^{*}+m\cdot \prod_{i=1}^{l^{\infty }-1}s_{i}\right)}{\sum_{m=-1}^{1}\sum_{n=-2}^{1}FMCC_{u+m,v+n}}$$

(6) $$x_{2}=\frac{\sum_{m=-1}^{1}\sum_{n=-2}^{1}FMCC_{u+m,v+n}\cdot \left(x_{2}^{*}+n\cdot \prod_{i=1}^{l^{\infty }-1}s_{i}\right)}{\sum_{m=-1}^{1}\sum_{n=-2}^{1}FMCC_{u+m,v+n}}$$

(7) $$y_{2}=\frac{\sum_{m=-1}^{1}\sum_{n=-2}^{1}FMCC_{u+m,v+n}\cdot \left(y_{2}^{*}+m\cdot \prod_{i=1}^{l^{\infty }-1}s_{i}\right)}{\sum_{m=-1}^{1}\sum_{n=-2}^{1}FMCC_{u+m,v+n}}$$

The location is achieved on low-resolution images. In order to restore the matching area to the sample *S*, we reversely scale up the refined position $\left(\left(x_{1}{,\;y}_{1}\right),\left(x_{2}{,\;y}_{2}\right)\right)$ on high resolution images.

### 3.2. Image Alignment Module

**Figure 3:**
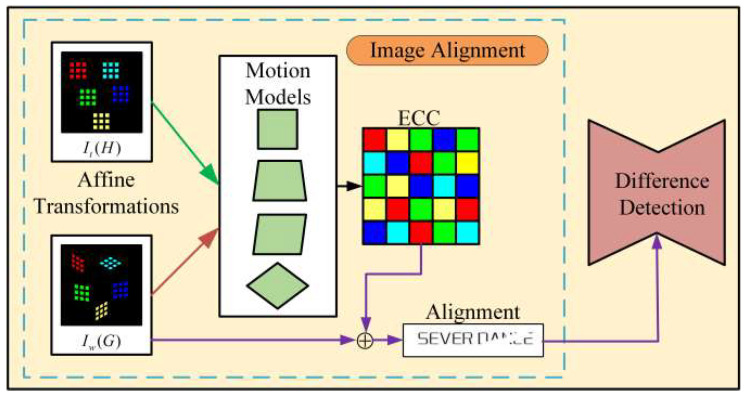
Schematic diagram of image alignment module.

After template matching, an image alignment module is introduced for defect detection. The implementation process is shown in Figure 3. Firstly, we propose image alignment to calibrate the matching region accurately, and then use the detection difference to complete the printing defect detection.

Since our template matching algorithm is performed on low-resolution images, there will be some positional deviations in the matching region. Therefore, it is necessary to use image alignment to modify the offset of the matching region. Image alignment searches the coordinate relationship between the pixels of the two images. If there is only an affine transformation between the two images, the problem of image alignment is transformed into the problem of solving the affine transformation matrix. In this paper, there is only a coordinate distortion between the matching region and the template, and thus image alignment can be regarded as a matrix estimation problem. We assume that the template is recorded as *I_t_*(*H*), the corresponding pixel coordinate is *H* = (*x_t_*, *y_t_*), the region to be aligned is recorded as *I_w_*(*G*), and the corresponding pixel coordinate is *G* = (*x_w_*, *y_w_*). Then, the image alignment only needs to search the transformation relationship between *H* and *G*, that is *G* = β(H; p), where **p** = (*p*_1_, …, *p_n_*)^T^. Finally, the alignment problem can be converted into an estimation of the parameter **p**.

(8) $$I_{t}(H)=I_{w}(\beta (H;p))$$

Assuming that there are *K* central coordinates between the alignment region and the template, the alignment region is composed of template vector ***i****_t_* and warped vector ***i****_w_*, which are defined as follows:

(9) $$i_{t}={\left[I_{t}\left(H_{1}\right)I_{t}\left(H_{2}\right)\cdots I_{t}\left(H_{K}\right)\right]}^{T}$$

(10) $$i_{w}(p)={\left[I_{w}\left(G_{1}(p)\right)I_{w}\left(G_{2}(p)\right)\cdots I_{w}\left(G_{K}(p)\right)\right]}^{T}$$

Then, the alignment metric enhanced correlation coefficient (*ECC*) is defined to calculate the motion transformation matrix and realize the alignment of the warped image:

(11) $$ECC={\left\|\frac{{\bar{i}}_{t}}{\left\|{\bar{i}}_{t}\right\|}-\frac{{\bar{i}}_{w}(p)}{\left\|{\bar{i}}_{w}(p)\right\|}\right\|}^{2}$$

where ${\bar{i}}_{t}$ and ${\bar{i}}_{w}$ denote the zero-mean vectors of the template and warped vector, respectively.

After the alignment, the template and the alignment region are both sent to the detection difference module to extract the difference information. We perform adaptive threshold binarization on the template and the target image and introduce the structural similarity (SSIM) [31] to evaluate the similarity between the two binary images. It should be noted that we do not output the SSIM score, only the difference information. Then, we perform the defect contour search, and finally achieve the defect position.

## 4. Experimental Results and Analysis

In this section, we detail the experimental implementation process and evaluate the proposed printing defect detection method, including template matching performance and defect detection performance.

### 4.1. Evaluation Metrics

**Figure 4:**
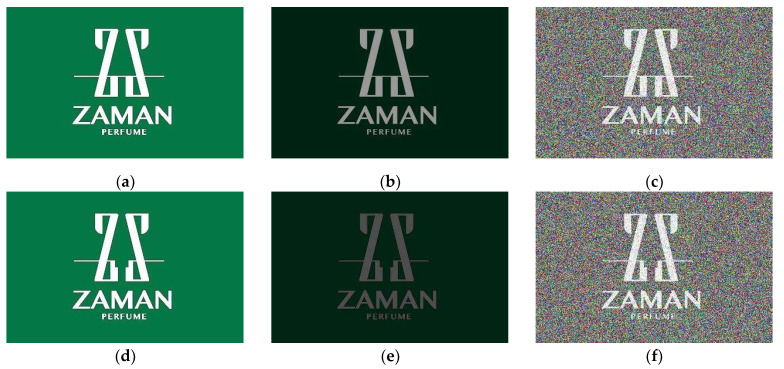
The examples of printing images: (**a**) zero-defect images; (**b**) zero-defect images with interference (illuminance); (**c**) zero-defect images with interference (noise); (**d**) defect images; (**e**) defect images with interference (illuminance); (**f**) defect images with interference (noise).

In order to verify the effectiveness of the proposed printing defect detection method, we collected 51 types of printing images with different sizes. Each type of printing image contained four different images, which were standard images without defect, called as zero-defect images (ZD), defect images (D), zero-defect images with interference (ZD_I), and defect images with interference (D_I). Examples of the printing images are shown in Figure 4. For the entire method, we used a confusion matrix to calculate several metrics for performance evaluation, including accuracy, precision, recall, F1-Score, and the area-under-curve (AUC). The confusion matrix is shown in Table 1.

Here, TP (true positive) means that the positive class in the sample is correctly predicted as a positive class, FP (false positive) means that the negative class in the sample is incorrectly predicted as a positive class, TN (true negative) means that the positive class in the sample is incorrectly predicted as a negative class, FN (false negative) means that the negative class in the sample is correctly predicted as a negative class. Simultaneously, we use the accuracy, precision, recall, F1-Score and area-under-curve (AUC) for performance evaluation [32]. In addition, in order to more intuitively reflect the specific detection performance on each type of sample, this paper used the true detection rate (TDR) and the false detection rate (FDR) as the evaluation metrics, which are defined as follows:

(12) $$\mathrm{TDR}=\frac{\mathrm{true}\;\mathrm{detection}\;\mathrm{number}}{\mathrm{defective}\;\mathrm{sample}}\times 100\%$$

(13) $$\mathrm{FDR}=\frac{\mathrm{false}\;\mathrm{detection}\;\mathrm{number}}{\mathrm{total}\;\mathrm{sample}}\times 100\%$$

To evaluate the performance of our proposed scale-adaptive template matching algorithm, we define the accuracy of template matching by adapting the measure of overlap area, which is shown as;

(14) $$\mathrm{IOU}=\frac{area\left(A_{P}\cap A_{GT}\right)}{area\left(A_{p}\cup A_{GT}\right)}$$

where *A_P_* and *A_GT_* represent the prediction box and the ground-truth box, respectively, and IOU reflects the overlap between the prediction results and the ground-truth results. The proportion of the overlap greater than a certain threshold (TH∈[0,1]) is used to achieve a continuous curve, and then the area under the curve (AUC) is calculated to quantify the accuracy of each method.

In addition, the experimental environment is Windows10 version 64-bit operating system, and all experiments are implemented on CPU (AMD Ryzen 75800H with Radeon Graphics 3.20 GHz). Therefore, our method has the advantage of low requirements for hardware equipment.

### 4.2. Experiment Results of Scale-Adaptive Template Matching

The proposed method consists of a scale-adaptive template matching module and an image alignment module. Moreover, the template matching performance will directly impact the defect detection results. Therefore, we first compared the performance of the proposed scale-adaptive template matching algorithm (FMCC) with several state-of-the-art methods, including SSD [13], ZNCC [14], SAD [15], and QATM [29].

In order to prove the effectiveness of our proposed method more reliably, we also conducted the experiment on a public dataset. During the experiment, we followed the evaluation protocol [20] and used the public dataset proposed by Wu et al. [33]. The dataset can be applied to different challenging situations, including deformation, illumination change, partial occlusion, and template matching. Meanwhile, the successful curves with varying IOU thresholds and AUC were used for quantitative comparison.

**Figure 5:**
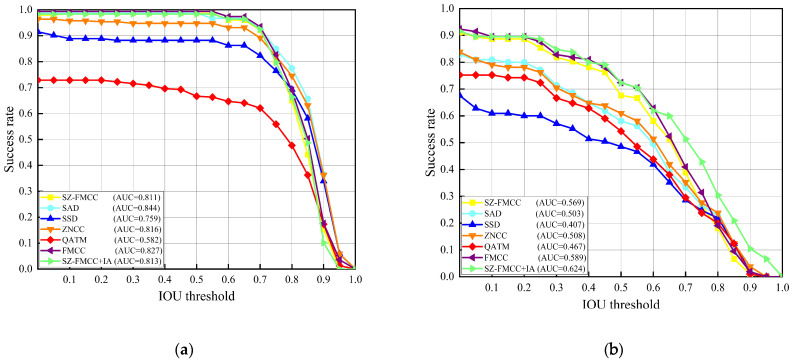
Quantitative analysis and comparison of the template matching performance. (**a**) Success curve based on AUC quantization accuracy on the collected printing dataset. (**b**) Success curve based on AUC quantization accuracy on the public dataset proposed in [33].

Firstly, we quantitatively analyzed the template matching method on the collected printing dataset. As shown in Figure 5a, the overall accuracy of FMCC method proposed in this paper was the highest, although the performance (0.844) of SAD is close to our performance (0.827). In addition, the matching method was implemented on the public dataset; the FMCC performance and all baseline methods performance are shown in Figure 5b. Again, our FMCC method achieved the best performance.

**Figure 6:**
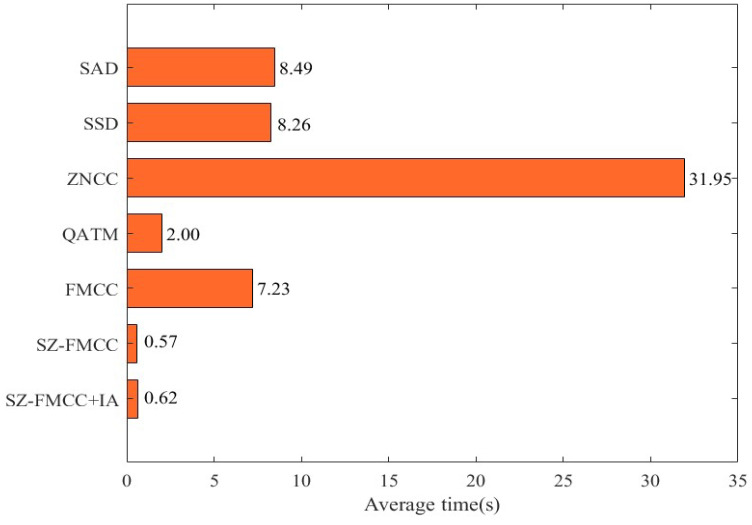
Time performance comparison on different models.

Then, we tested the time performance of these template matching algorithms on the collected printing dataset, and the results are shown in Figure 6. The execution time of our FMCC (7.23 s) is significantly faster than that of SAD (8.49 s), SSD (8.26 s), and ZNCC (31.95 s). Considering that the CNN-based feature extraction method is more complex than the sliding window method, we introduced a scale zooming factor to accelerate the implementation (SZ-FMCC). This method scales down the template and sample in equal proportions, which reduces parameter calculation. Therefore, the accelerated method (SZ-FMCC) can achieve a matching speed of 0.57 s; this is about 12 times than the proposed benchmark method (FMCC).

As shown in Figure 5 and Figure 6, we introduced a scale zooming factor to greatly accelerate the implementation (SZ-FMCC), but its AUC decreased to 0.811, which had a bad influence on defect detection. Therefore, we introduced an image alignment method to greatly improve the AUC of the method from 0.811 to 0.984 (SZ-FMCC+IA). Simultaneously, the execution time only increased from 0.57 s to 0.62 s.

**Figure 7:**
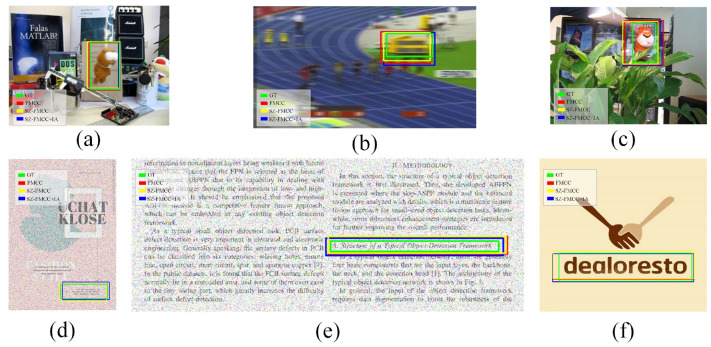
Performance evaluation of the scale-adaptive template matching algorithm proposed in this paper on partial samples; (**a**–**c**) sampled from the public dataset and (**d**–**f**) sampled from the collected dataset. (The ground-truth is labeled with a green box, and the matching results of FMCC, SZ-FMCC, and SZ-FMCC+IA are, respectively, labeled with red, blue, and yellow boxes).

In addition, Figure 7 shows the matching results of the different challenging situations. It can be seen that the matching regions of the proposed method almost overlapped with the labeled ground-truth regions.

At the same time, in order to more intuitively illustrate the performance, we calculated the IOU of the predicted box and the ground-truth box. As shown in Table 2, it can be seen that the IOU of SZ-FMCC is lower than FMCC. However, the IOU of SZ-FMCC+IA increased significantly after introducing the alignment method, indicating that our proposed method improves the accuracy of template matching effectively.

### 4.3. Experiment Results of Printed Matter Defect Detection

In order to validate the detection performance, we tested the proposed method on the printing dataset. Table 3 shows the different evaluation values, including the accuracy, precision, recall, F1-Score, and AUC. As shown in Table 3, the accuracy rate of the defect detection method proposed in this paper reached 93.62%, indicating that the model has a high probability of correct identification of defects, and the recall rate was as high as 100%, which means that the model can detect all defects in the actual sample and meets the actual industrial detection requirements. Moreover, the F1-Score, as the harmonic mean of precision and recall, can reached 94.01%. Similarly, the AUC also comprehensively reflected the overall satisfactory performance.

**Figure 8:**
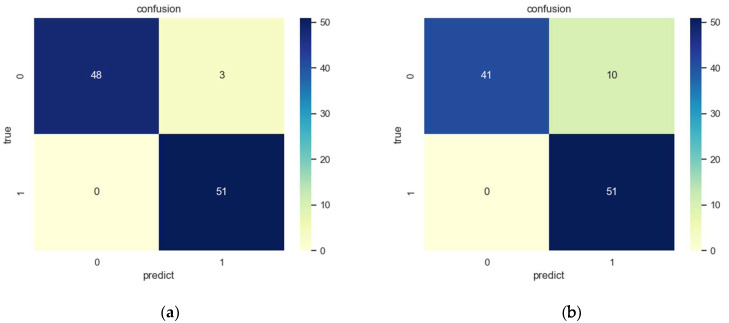
Confusion matrices on printed datasets; (**a**) the confusion matrix on the images without interference, and (**b**) the confusion matrix on the images with interference.

Meanwhile, in order to discuss the impact of interference, the confusion matrices on different types are shown in Figure 8. Obviously, the introduction of confusion matrices has shown that the false detections are generally from samples with interference. This is mainly because interference may generate false defects, which have an influence on the detection results.

Table 4 shows the effect of the model on each sample type. The proposed printing defect detection method has a TDR of 96.07% for zero-defect images (ZD), and a TDR of 94.12% for defect images (D). Although the false detection mainly results from the samples with interference (ZD_I and D_I), the method has high anti-interference ability in general.

**Figure 9:**
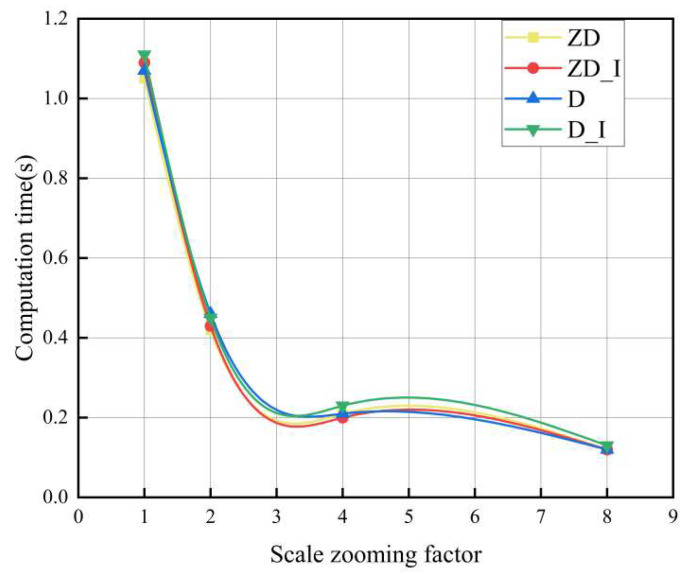
Computation time with respect to the scale zooming factor.

In order to verify the effect of the scale zooming factor *Z* = 2*^r^* (*r* = 0,1,2……) on the detection speed as *r* increases, we randomly selected some printing images to test the performance. The changing trend between scale zooming factor and detection speed is shown in Figure 9.

It can be seen from Figure 9 that as the scale zooming factor *r* increases, the detection time tends to decrease. Furthermore, these curves are almost the same, indicating that our method has high robustness.

## 5. Conclusions

In this paper, we propose a novel printing defect detection method based on scale-adaptive template matching and image alignment. Simultaneously, we introduce a new similarity measurement metric called feature map cross-correlation (FMCC), for improving the accuracy of similarity measurements. Our method extracts the underlying features of the template and the target image through scale-adaptive depth-wise convolution, and then optimizes that on the basis of FMCC. The method greatly improves the running speed while ensuring detection accuracy. The experimental results demonstrate that the method can quickly and accurately find the location of the defect. At the same time, it is also proven that our method achieves state-of-the-art defect detection performance with strong real-time detection and anti-interference performance.

The limitation of this work is that the parameters of the input model were obtained through a large number of trials. Indeed, these parameters can be determined by the underlying features of the image. In the future, we will continue to optimize the method by designing appropriate adaptive input parameters for each module. Meanwhile, we can conduct research in the field of reinforcement learning. In addition, we will apply the proposed method to defect detection of more industrial products. 

## Figures and Tables

**Table 1:** Confusion matrix.

	Positive	Negative
Positive	TP	FP
Negative	FN	TN

**Table 2:** The IOU of the ground-truth box and the prediction box on partial images of the printing dataset.

Image	FMCC	SZ-FMCC	SZ-FMCC+IA
Figure 7a	0.910	0.839	0.845
Figure 7b	0.791	0.663	0.753
Figure 7c	0.534	0.523	0.534
Figure 7d	0.887	0.754	0.926
Figure 7e	0.569	0.563	0.586
Figure 7f	0.807	0.791	0.907

**Table 3:** Defect detection performance evaluation.

Accuracy (%)	Precision (%)	Recall (%)	F1 (%)	AUC (%)
93.62	88.69	100.00	94.01	94.00

**Table 4:** Evaluation values of different sample types.

Sample Types	TDR (%)	FDR (%)
ZD	96.07	0.98
ZD_I	78.43	5.39
D	94.12	1.47
D_I	92.16	1.96

## Data Availability

Not applicable.
